# An Unusual Cause of Buttock Pain in a Collegiate Football Player: A Septic Sacroiliac Joint

**DOI:** 10.7759/cureus.100835

**Published:** 2026-01-05

**Authors:** Jonathan R Guin, Ryan Moran, Brett C Bentley

**Affiliations:** 1 Family Medicine, University of Alabama, Tuscaloosa, USA; 2 Athletic Training, University of Alabama, Tuscaloosa, USA

**Keywords:** college sports, football injury, primary care sports medicine, sacroiliac infection, septic sacroiliitis

## Abstract

Early recognition of sacroiliac (SI) joint infection can be challenging in young, otherwise healthy individuals presenting with nonspecific buttock or thigh pain. We present the case of a 21-year-old collegiate football player who developed progressive buttock and thigh pain after a minor fall during practice. Initial evaluation suggested sciatic nerve compression, and early MRI revealed only subtle soft tissue edema. Despite conservative treatment and a nerve block, his pain acutely worsened, and he developed fever and chills. Repeat MRI five days later demonstrated a fluid collection consistent with septic sacroiliitis and abscess formation, with additional findings of iliopsoas and gluteal myositis. Blood cultures and CT-guided aspiration confirmed methicillin-sensitive *Staphylococcus aureus* (MSSA). The patient was treated with intravenous nafcillin for four weeks via peripherally inserted central catheter (PICC) line following a nine-day hospital admission. He made a full recovery and started a slow return to play back into collegiate football three months after admission. This case highlights the diagnostic challenge of septic sacroiliitis in young athletes, the importance of repeat MRI when symptoms progress, and the favorable prognosis when prompt, culture-directed antibiotic therapy is initiated.

## Introduction

Septic arthritis of the sacroiliac (SI) joint is a rare clinical diagnosis, accounting for only 1-2% of septic arthritis cases [1]. Its rarity and vague clinical presentation frequently result in diagnostic delay, which can increase morbidity [2]. Reported time to diagnosis often ranges from one to three weeks, with some series citing delays of over a month before appropriate imaging or microbiologic confirmation is obtained [2-3]. Risk factors include comorbid chronic conditions such as diabetes and rheumatologic disease, age greater than 80 years old, intravenous drug use, and pregnancy [2-3]; however, the condition may also occur in otherwise healthy individuals.

The SI joint is a synovial articulation that connects the sacrum to the ilium, reinforced by strong anterior and posterior ligaments and supported by limited movement of only a few degrees. It bears substantial axial load transfer between the spine and lower extremities, while its deep anatomic position and complex innervation from the lumbosacral plexus make localization of pain challenging. As a result, inflammation or infection of the joint can produce pain radiating to the buttock, posterior thigh, or groin, often resembling other musculoskeletal disorders.

In athletes, sacroiliitis is particularly uncommon, with only sporadic cases described in the literature. Estimates suggest that infection-related sacroiliitis represents less than 0.5% of cases presenting with low back or buttock pain in young active populations [4]. When it does occur, it may follow trauma, hematogenous spread after minor skin or soft tissue injury, or iatrogenic inoculation from injections [3,5]. Because of overlapping clinical features, it can closely mimic several musculoskeletal conditions, including lumbar disc herniation, piriformis syndrome, SI dysfunction, hamstring strain, or lumbosacral radiculopathy [4].

Imaging plays a critical role: plain radiographs are often normal early, while magnetic resonance imaging (MRI) provides the highest sensitivity for detecting edema, effusion, and early abscess formation [5]. Diffusion-weighted MRI may further increase diagnostic confidence [6].

We present the case of a collegiate football player with septic sacroiliitis due to methicillin-sensitive *Staphylococcus aureus *(MSSA). His course highlights the overlap with musculoskeletal injuries, the importance of repeat MRI in evolving cases, and the role of culture-directed antibiotic therapy in achieving full recovery.

## Case presentation

A collegiate football player in his early 20s (height 74 inches, weight 175 pounds, BMI 24.5 kg/m²) sustained a minor fall onto his buttock during practice. He was otherwise healthy, with no chronic medical conditions, no family history of autoimmune or infectious joint disease, and no recent infections or pertinent musculoskeletal injuries. He does not take any medications, and he reported no allergies, alcohol, tobacco, or drug use. He was able to complete practice but developed worsening buttock and left thigh pain six days later. Due to worsening pain after hours, he presented to a local emergency department, where plain radiographs were unremarkable. While in the emergency department, he did have placement of a peripheral intravenous line placed for medications and fluids. He was later discharged home from the emergency department with an opiate pain medication, non-steroidal anti-inflammatory drug (NSAID), corticosteroid, and a muscle relaxant.

The following morning, he presented to our clinic with persistent pain and was diagnosed clinically with sciatic nerve compression secondary to trauma, with associated inflammatory response and possible hematoma formation. His exam was remarkable for moderate tenderness to palpation over his left sacrum and piriformis area, slightly decreased strength with left knee flexion (4+/5) compared to the right (5/5), and decreased Achilles reflex on the left (1+) compared to the right (2+). It was recommended that he discontinue the opiate pain medication and NSAID while continuing the corticosteroid and muscle relaxant. An MRI of the pelvis with and without contrast was obtained to evaluate the sciatic nerve, given his exam findings, and an MRI of the lumbar spine without contrast was also ordered to rule out acute lumbar pathology. He was restricted from sports participation pending further evaluation.

**Figure 1:**
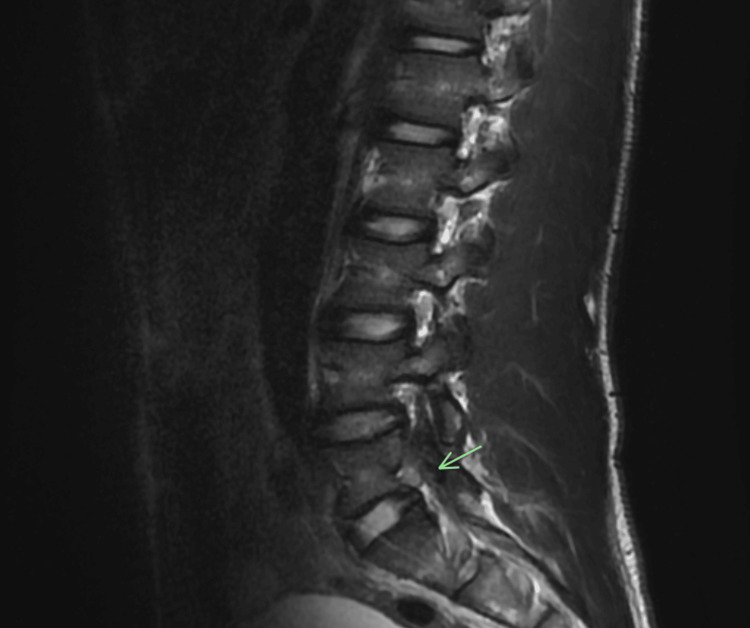
Sagittal T2-weighted MRI of the lumbar spine demonstrating moderate left-sided neural foraminal narrowing at the L5–S1 level. The green arrow indicates foraminal narrowing at L5/S1.

**Figure 2:**
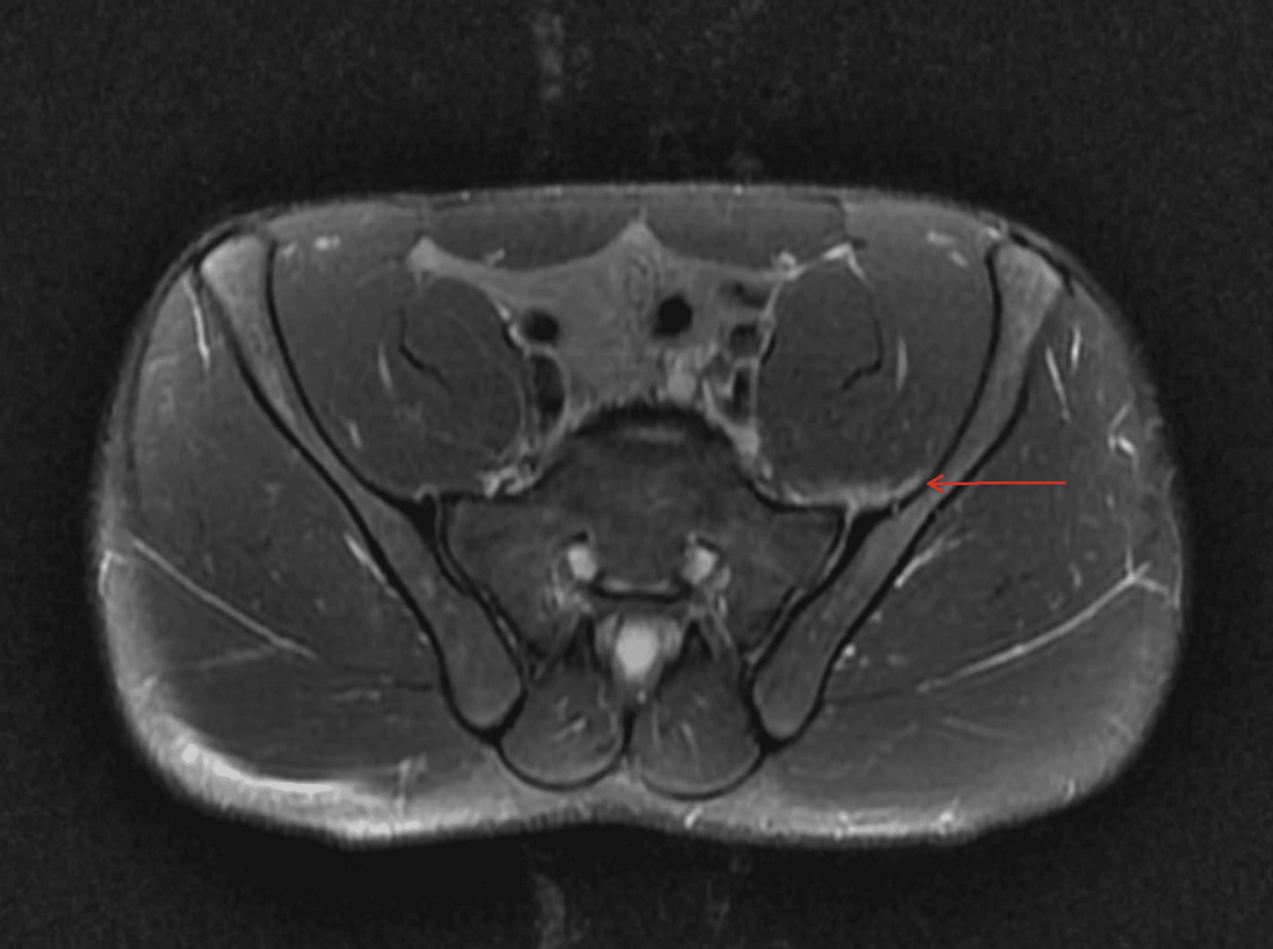
Axial T2-weighted MRI of the pelvis, with and without contrast, demonstrating soft tissue edema anterior to the left sacroiliac joint along the deep fibers of the iliopsoas muscle. The red arrow indicates the site of pathology.

MRI of the lumbar spine revealed moderate neural foraminal narrowing on the left at L5/S1 (Figure 1), which could explain his symptoms. MRI of the pelvis demonstrated soft tissue edema anterior to the left SI joint along the deep fibers of the iliopsoas muscle without overlying hematoma or gross abnormality near the sciatic nerve (Figure 2). On the following day, he received a left L5 nerve block at another facility.

He returned to our clinic five days later, now in a wheelchair secondary to pain, with excruciating buttock pain, subjective fever, and chills. Vital signs showed blood pressure 100/78 mmHg, pulse 79 bpm, oxygen saturation 98% on room air, respiratory rate 17 bpm, and temperature 99.7°F (37.6°C). Physical exam was largely limited due to severe pain but revealed marked tenderness to palpation of the left buttock; strength testing of the extensor hallucis longus was 4+/5 on the left compared to 5/5 on the right. Complete blood count (CBC), erythrocyte sedimentation rate (ESR), and C-reactive protein (CRP) were obtained. The CBC showed a normal white blood cell (WBC) count of 7.8 cells/mm³ (normal 5-10.0 cells/mm³), but did show an elevated neutrophil percentage at 76.9% (normal 50.0-64.0%), which could be suggestive of a bacterial infection. ESR and CRP were both elevated at 32 mm/hr (normal 0-15 mm/hr) and 232 mg/L (normal 0-10 mg/L), respectively, indicating a high inflammatory response. Given this acute decompensation, a repeat MRI was urgently obtained with concern for infection.

**Figure 3:**
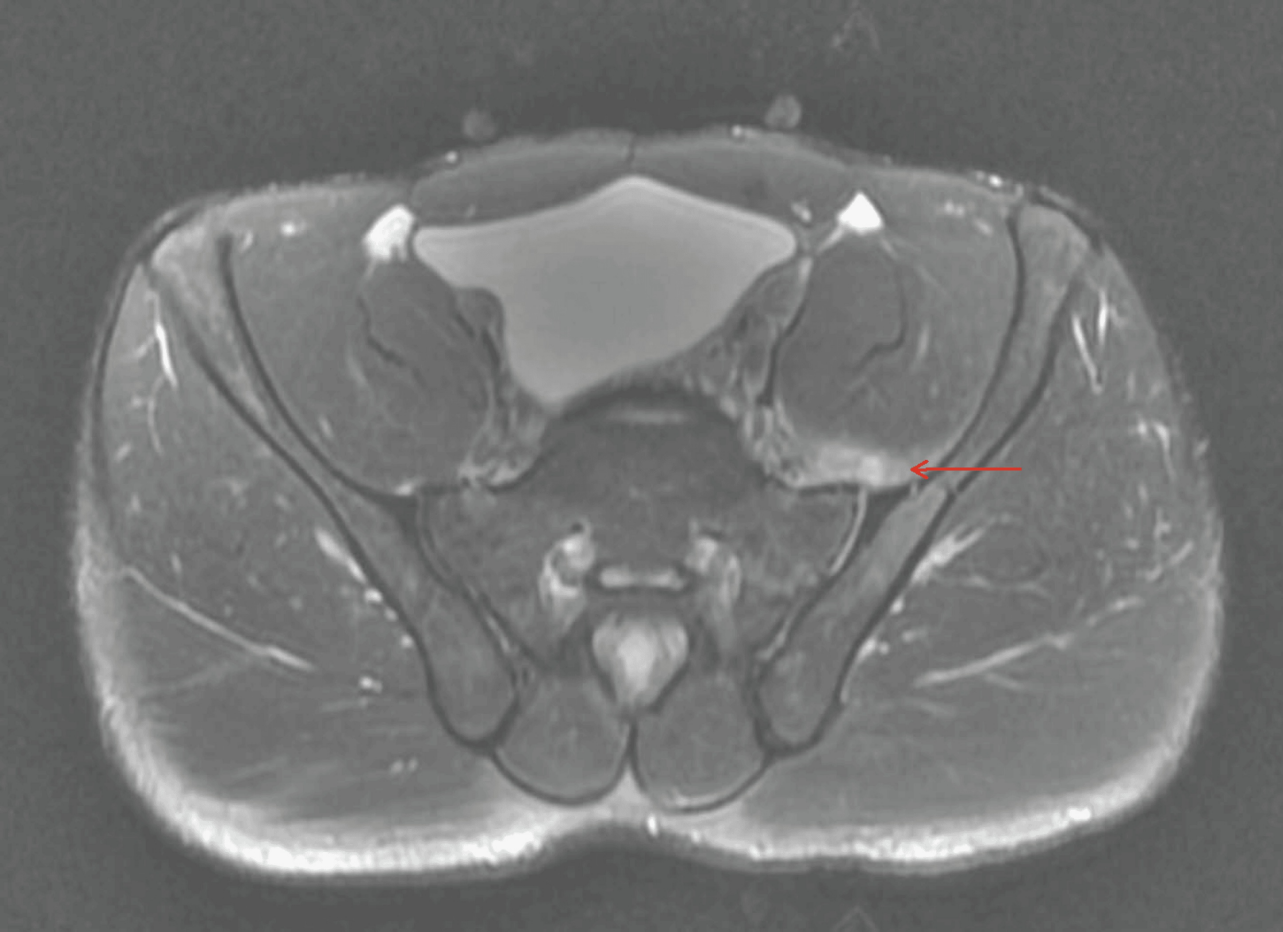
Axial T2-weighted MRI of the pelvis, with and without contrast, demonstrating increased edema at the left sacroiliac joint and a central fluid collection consistent with septic arthritis and a small abscess. The red arrow indicates the site of pathology.

Repeat MRI revealed interval worsening with edema in the SI joint and a 1.8 × 0.9 × 1.3 cm fluid collection anterior to the left SI joint, consistent with septic arthritis with small abscess (Figure 3). Edema was again noted along the deep fibers of the iliopsoas, extending through the sciatic notch and deep to the gluteus maximus muscle. The gluteus minimus and proximal vastus lateralis also demonstrated edema consistent with myositis.

He was admitted for intravenous antibiotics and orthopedic surgery consultation. Blood cultures revealed methicillin-sensitive Staphylococcus aureus (MSSA) bacteremia (Table 1). Repeated labs while inpatient showed a persistently normal WBC, while ESR remained elevated throughout his stay from 31 to 68 mm/hr. Echocardiogram showed no intracardiac vegetations or abscess as a potential source of infection. CT-guided aspiration of the left SI joint also isolated MSSA (Table 2). Orthopedic surgery determined that the abscess was not amenable to drainage. Infectious diseases require four weeks of intravenous antibiotics. The patient was discharged nine days later with a peripherally inserted central catheter (PICC) line for outpatient nafcillin infusions.

**Table 1:** Blood culture results with antibiotic susceptibility testing

Blood culture	
Result	Staphylococcus aureus
Antibiotic susceptibility testing	
Oxacillin	Susceptible
Vancomycin	Susceptible

**Table 2:** Left sacroiliac joint aspirate culture results with antibiotic susceptibility testing

Left sacroiliac joint aspirate culture	
Result	Moderate *Staphylococcus aureus*
Antibiotic susceptibility testing	
Amoxicillin/clavulanic acid	Susceptible
Ceftriaxone	Susceptible
Clindamycin	Susceptible
Erythromycin	Susceptible
Gentamicin	Susceptible
Oxacillin	Susceptible
Tetracycline	Susceptible
Trimethoprim/sulfamethoxazole	Susceptible
Vancomycin	Susceptible

The patient continued to improve clinically and was cleared to begin a gradual return to sport six weeks after hospital discharge, ultimately returning to full collegiate football participation after three months. Table 3 shows the full timeline and clinical summary.

**Table 3:** Clinical timeline and summary of events MSSA: Methicillin-sensitive *Staphylococcus aureus, *PICC: peripherally inserted central catheter, IV: intravenous, CBC: complete blood count, WBC: white blood cell, ESR: erythrocyte sedimentation rate, CRP: C-reactive protein

Day	Event / clinical presentation	Investigations and findings	Management / outcome
Day 0 (Injury)	Minor fall onto buttock during football practice; completed practice without limitation.	—	No immediate intervention.
Day 6	Progressive buttock and left thigh pain develops; presents to local emergency department.	X-rays unremarkable.	Discharged with opiate, NSAID, corticosteroid, and muscle relaxant.
Day 7	Persistent pain; clinic evaluation reveals tenderness over left sacrum/piriformis and mild weakness; diagnosed with sciatic nerve compression.	MRI lumbar/pelvis ordered; neurological findings documented.	Stopped opiate/NSAID; continued corticosteroid and muscle relaxant; restricted from sport.
Day 8	MRI lumbar: foraminal narrowing; MRI pelvis: mild soft tissue edema near left sacroiliac joint.	MRI lumbar spine: L5/S1 foraminal narrowing; MRI pelvis: mild edema, no hematoma.	Referred for left L5 nerve block at outside facility.
Day 9	Left L5 nerve block performed	—	Left L5 nerve block performed without complication.
Day 13	Returns to clinic in wheelchair with severe pain, subjective fever, and chills.	CBC: normal WBC (7.8), neutrophils 76.9%; ESR 32 mm/hr; CRP 232 mg/L.	Urgent repeat MRI ordered with concern for infection.
Day 13 (same day)	Repeat MRI shows sacroiliac joint edema and small abscess consistent with septic sacroiliitis.	Repeat MRI: sacroiliac joint abscess 1.8×0.9×1.3 cm; iliopsoas/gluteal myositis.	Hospital admission for IV antibiotics and orthopedic consultation.
Hospital Days 1–9	Inpatient management; blood and joint aspirate cultures positive for MSSA; IV nafcillin initiated; discharged after 9 days with PICC for 4 weeks IV therapy.	Blood cultures: MSSA; ESR 31–68 mm/hr; echocardiogram negative; CT-guided aspiration positive for MSSA.	Continued IV nafcillin; abscess not drained; discharged with PICC line.
6 Weeks Post-discharge	Gradual return to sport initiated.	—	Gradual rehabilitation and monitored return to play.
3 Months Post-admission	Full recovery; cleared for return to collegiate football.	—	Full return to collegiate football participation.

## Discussion

Septic sacroiliitis is an uncommon but potentially devastating cause of buttock and low back pain. Diagnosis is often delayed due to its rarity and nonspecific clinical presentation. Typical features include localized buttock, low back, or thigh pain, sometimes accompanied by systemic symptoms such as fever or chills [2,4]. In athletes, the overlap with more common musculoskeletal diagnoses-such as muscle strain, lumbar nerve root impingement, piriformis syndrome, or SI joint dysfunction-further complicates recognition [4].

Diagnostic considerations

Plain radiographs are frequently normal in early disease [5]. MRI is the preferred imaging modality, with high sensitivity for detecting joint effusion, marrow edema, and soft tissue involvement [5]. Diffusion-weighted MRI has also been shown to improve diagnostic confidence in subtle cases [6]. In this patient, the initial lumbar MRI revealed moderate left-sided foraminal narrowing, which initially suggested a mechanical etiology and contributed to a short diagnostic delay. The subsequent pelvic MRI demonstrated only mild soft tissue edema along the iliopsoas, findings that were interpreted as post-traumatic inflammation rather than infection. Repeat MRI five days later, however, showed a new periarticular fluid collection with myositis, findings diagnostic of septic sacroiliitis with abscess formation. This progression highlights the importance of re-evaluation and repeat imaging when pain worsens or systemic symptoms develop despite an initially nondiagnostic study.

Etiology and risk factors

This patient had no underlying medical comorbidities, immunosuppression, or classic risk factors. The exact etiology was not identified. However, possible sources include iatrogenic introduction of bacteria during his initial emergency department treatment, which included intravenous line placement, or from the L5 nerve block performed at an outside facility approximately five days later. The L5 nerve block was documented as performed under standard aseptic precautions, yet transient bacteremia following either intervention remains a plausible source of inoculation given the close temporal relationship between these procedures and his subsequent systemic symptoms.

While direct trauma (as in this case) is rarely a sole cause of infection, it may create a local environment conducive to bacterial seeding through microvascular disruption or hematoma formation. In addition, unrecognized skin breaches sustained during athletic activity could have provided a route for hematogenous spread. The isolation of methicillin-sensitive Staphylococcus aureus (MSSA) is consistent with the most frequently reported pathogen in pyogenic sacroiliitis and supports a potential skin or procedural source [1,2,5].

Management

First-line treatment involves prolonged intravenous antibiotics, typically for four to six weeks. Surgical drainage or debridement is considered in cases with large, accessible abscesses or poor response to medical management [7,8]. Although most patients recover fully with antibiotic therapy alone, approximately 10-20% may require surgical intervention for abscess drainage or joint stabilization when infection extends beyond the joint capsule [8]. Multidisciplinary management involving infectious disease, radiology, and orthopedic specialists is critical to ensure complete resolution and prevent chronic complications. In this case, despite the presence of a small abscess, orthopedic surgery deemed it not amenable to drainage. Blood cultures and CT-guided aspiration confirmed MSSA, guiding the use of targeted intravenous nafcillin.

Outcomes

Prompt initiation of antibiotics is associated with favorable outcomes [5,8], while delayed recognition increases the risk of fulminant sepsis, pelvic instability, or chronic pain [7,8]. Reported recovery rates in published series range from 80-90%, particularly among young and otherwise healthy individuals [5,8]. Athletes often achieve full return to play with appropriate, culture-directed antibiotic therapy and gradual rehabilitation. Despite the diagnostic challenge, careful reassessment, repeat MRI, and targeted antibiotic management led to full recovery in this patient, who successfully completed a structured return-to-play protocol three months after discharge.

## Conclusions

Septic sacroiliitis is a rare but serious condition that can masquerade as common musculoskeletal injuries, particularly in young athletes. Clinicians should maintain a broad differential when evaluating refractory buttock and low back pain, especially when symptoms worsen or systemic features emerge. MRI is the imaging modality of choice and repeat studies may be necessary if initial results are inconclusive. Early recognition, culture confirmation, and intravenous antibiotic therapy are essential to prevent complications and enable full recovery.
